# The Content of Phenolic Compounds and Mineral Elements in Edible Nuts

**DOI:** 10.3390/molecules27144326

**Published:** 2022-07-06

**Authors:** Magdalena Woźniak, Agnieszka Waśkiewicz, Izabela Ratajczak

**Affiliations:** Department of Chemistry, Faculty of Forestry and Wood Technology, Poznan University of Life Sciences, Wojska Polskiego 75, 60625 Poznan, Poland; agnieszka.waskiewicz@up.poznan.pl (A.W.); izabela.ratajczak@up.poznan.pl (I.R.)

**Keywords:** nuts, mineral elements, phenolic compounds, CAPE

## Abstract

Edible nuts are an important component of a healthy diet, and their frequent consumption has beneficial impact on human health, including reducing the risk of cardiovascular and neurodegenerative diseases. Moreover, various factors, including cultivar, climate, soil characteristic, storage and treatment have influence on the chemical composition of nuts. Therefore, nine tree nut types and peanuts commonly available on Polish market were evaluated for phenolic profile and mineral elements content. The concentration of individual phenolic compounds, including flavonoids, aromatic acids and caffeic acid phenethyl ester (CAPE) was determined by ultra-high pressure liquid chromatography, while the content of macro-elements and trace minerals was analyzed by atomic absorption spectrometry. The phenolic profile of analyzed nuts substantially varied depending on the type of nut. The highest total content of all analyzed flavonoids was determined in walnuts (114.861 µg/g), while the lowest in almonds (1.717 µg/g). In turn, the highest total content of all tested aromatic acid was determined in pecans (33.743 µg/g), and the lowest in almonds (0.096 µg/g). Epicatechin and cinnamic acid were detected in the highest concentration in tested nuts. Moreover, in examined nuts (except walnuts and Brazil nuts), the presence of CAPE was confirmed. The tested nuts were also characterized by wide variation in element concentrations. Almonds contained high concentration of macro-elements (13,111.60 µg/g), while high content of trace elements was determined in pine nuts (192.79 µg/g). The obtained results indicate that the tested nuts are characterized by a significant diversity in the content of both phenolic compounds and minerals. However, all types of nuts, apart from the well-known source of fatty acids, are a rich source of various components with beneficial effect on human health.

## 1. Introduction

Edible nuts are an important component of a healthy diet, and their consumption is increasing worldwide as more and more people recognize the need for a healthy lifestyle [1,2]. The most consumed nuts in the world include, among others almonds, Brazil nuts, pecans, walnuts, cashews, macadamia nuts, hazelnuts, pine nuts and pistachios [3]. Peanuts are also very popular, although they belong to legumes, due to the similar nutritional composition to nuts, they are classified by consumers and nutritionists in this group of products [4]. Literature data and clinical trials suggest that frequent nut consumption has a beneficial impact on human health, including reducing risk of cardiovascular diseases, obesity, type-II diabetes, various types of cancer or neurodegenerative diseases [2,4,5,6,7,8]. Moreover, nuts exhibited antioxidant and antimicrobial properties, including activity against *Staphylococcus aureus*, *Bacillus cereus*, *Escherichia coli* or *Klebsiella pneumonia* [9,10,11].

The beneficial effect of nut consumption on human health is related to the diversified chemical composition. Of course, the exact chemical composition of the nuts varies according to the type. In addition, literature reports indicated that various factors, including cultivar, climate, soil characteristic, storage and treatment (e.g., temperature and roasting time), influence the chemical composition of nuts [9,12,13,14]. However, in general, nuts are known as a rich source of unsaturated fatty acids and proteins [15]. Nuts, with the exception of chestnuts, which contain low level of fats, have a high total fat content, ranging from 46% (cashews, pistachios) to 76% (macadamia nuts) [16]. Among unsaturated fatty acids, oleic, linoleic and α-linolenic acids are the prominent in all types of nuts [16,17]. In addition, nuts are good source of phytochemicals, including vitamins (e.g., niacin, tocopherols and folic acid) and minerals, such as calcium, magnesium, potassium, selenium or iron [16,18]. Macro-elements have many functions in the human body, including the initiation of the production of hormones (along with vitamins) or accelerate metabolic processes [19]. In turn, trace elements are components of hormones and enzymes, and are involved in immune regulation, nerve conduction or muscle contractions [19,20]. According to literature reports, various types of nuts contain both macro- and microelements, including Ca, Mg, K, Zn, Cu, Mn and Fe [1,18]. In addition, Brazil nuts are a rich source of selenium, and one Brazil nut may contain up to 400 μg of Se [21].

Phenolic compounds are a group of nut components that may be considered to be a major phytochemicals for health benefits and characterized by wide pharmacological activity, including anticancer, antioxidant, anti-inflammatory, antimicrobial and antiviral [22,23,24,25]. The regular intake of polyphenols may prevent cardiovascular and neurodegenerative diseases and also reduce the risk of diabetes and several other diseases and physiological syndromes [22,24,26]. In different types of nuts, various individual phenolic compounds have been identified. However, the most commonly determined phenols in nuts are gallic acid, ellagic acid and catechin [9,14,27]. Among flavonoids in nuts, epicatechin, quercetin, naringenin, kaempferol, isothamnetin, galangin and apigenin have also been identified [14,27,28,29]. In turn, aromatic acids identified in nuts include caffeic, coumaric, ferulic, vanillic and syringic acids [3,14,29].

The aim of this study was to determine the phenolic profile and the content of mineral elements in nuts. Nine commonly consumed tree nuts, namely pecans, walnuts, cashews, macadamia nuts, hazelnuts, almonds, Brazil nuts, pistachios and pine nuts and peanuts, available on the Polish market were used for the research. In nut samples, the concentration of individual phenolic compounds, including flavonoids, aromatic acids and caffeic acid phenethyl ester (CAPE), was determined by ultra-high pressure liquid chromatography. In turn, the content of mineral elements, including macro-elements and trace minerals in nuts was determined by atomic absorption spectrometry. To the best of our knowledge, this article is the first to report on the phenolic profile and mineral content of nuts available on the local market. In addition, the results could expand existing knowledge concerning the chemical composition and presence of phytochemicals in nuts and may be of interest to various groups of scientists, including biochemists, food scientists and nutritionists.

## 2. Results

### 2.1. Concentration of Phenolic Compounds

The concentrations of phenolic compounds, including flavonoids, phenolic acids and caffeic acid phenethyl ester in common edible nuts available on Polish market are presented in Table 1 and Table 2.

The phenolic profile of analyzed nuts was very varied depending on the type of nut. Pecan nuts were characterized by the greatest diversity of identified flavonoids—9 out of 12 analyzed flavonoids were determined in this type of nuts. In contrast, only three flavonoids were identified in Brazil nuts—epicatechin, pinostrobin and galangin. The highest total concentration of all analyzed flavonoids was determined in walnuts and amounted to 114.861 µg/g. The studies by Yang et al. showed that among the ten examined types of nuts, walnuts were characterized by the highest total flavonoid content [7]. In contrast, the lowest total amount of flavonoids was analyzed in almonds and amounted to 1.717 µg/g. All nut samples (except almonds) were characterized by the highest content of epicatechin, from 114.296 µg/g (walnuts) to 1.162 µg/g (pine nuts). The most abundant phenol in walnuts was epicatechin, which constituted about 85% of the content of all determined flavonoids. Epicatechin is one of the most abundant plant phenols in human diet with prominent biological properties, such as antioxidant, anti-inflammatory, antitumor and anti-diabetic [30,31,32]. Moreover, epicatechin has beneficial effect on nervous system and enhances muscle performance, improves cardiac function following protect the cardiovascular system [30,31,33]. The concentration of catechin was also detected at a high level in six among ten analyzed nuts, with concentrations from 0.722 µg/g for almonds to 22.263 µg/g for cashew nuts. Catechin is characterized by outstanding pharmacological effects, including anticancer, antioxidant, anti-inflammatory, hepatoprotective, anticoagulant, antihypertensive, anti-arthritis, antidiabetic, neuroprotective and memory-enhancing properties [34,35]. Moreover, catechin isolated from cashew nut shells showed activity against clinical isolates of MRSA (methicillin-resistant *Staphylococcus aureus*) [36].

Galangin was the only flavonoid identified in all examined nut samples. Its highest concentration was detected in macadamia nuts (2.276 µg/g) and the lowest content in pistachios (0.007 µg/g). Literature data describe galangin as a strong anticancer and antimicrobial agent. The molecular pathways of this flavonoid are involved in suppressing various malignancies, including osteosarcoma and cancer of the lung, stomach and liver [23,37,38]. In turn, antimicrobial activity of galangin involved various strains of bacteria and fungi, such as *S. aureus*, *E. coli*, *K. pneumonia*, *Bacillus subtilis* and *Candida albicans* [39,40,41]. Moreover, besides activity against amoxicillin-resistant *E. coli* and penicillin-resistant *S. aureus*, galangin was able to reverse bacterial resistance to antibiotics [42,43]. In most of the tested nut samples, trace amounts of apigenin, kaempferol and pinostrobin were also determined. Only, the concentration of pinostrobin, which showed that biological activity, such as anticancer and antiviral, was detected in pecan nuts at higher level (6.355 µg/g) than other nuts [44]. Additionally, the amount of apigenin and kaempferol in pecan nuts was higher than in other samples. The remaining analyzed flavonoids were found in less than half of the nuts and were detected in trace amounts.

The content of aromatic acids (Table 2) in nuts, both in terms of quality and quantity, was very diversified. The most frequently determined aromatic acid, which was identified in nine out of ten tested nuts (except walnuts) was sinapic acid, and its concentration ranged from 0.050 µg/g for macadamia nuts to 10.373 µg/g for pecan nuts. Sinapic acid is known for its biological properties, including antioxidant, anti-inflammatory, anticancer and antimicrobial activity [45,46]. In at least half of the analyzed nuts various concentrations of vanillic, syringic, caffeic and cinnamic acids were detected. In turn, hydroxycinnamic acid was identified in trace amounts only in two types of nuts—hazelnuts and pine nuts. The highest total concentration of all analyzed aromatic acids was determined for pecan nuts and amounted to 33.743 µg/g. In turn, the lowest total content of analyzed acids was determined in almonds and was 0.096 µg/g. In addition, only three acids were identified in almonds: caffeic, sinapic and ferulic. The highest concentration among analyzed acids was detected for cinnamic acid in pecan nuts and was 12.774 µg/g. All of determined aromatic acids in nuts are characterized by biological activity, including neuroprotective activity (caffeic, ferulic, sinapic, coumaric and syringic acids), antioxidant activity (caffeic, cinnamic, ferulic and vanillic acids), hepatoprotective effect (syringic acid), antitumor activity (cinnamic acid) and antiviral action (caffeic and coumaric acids) [47,48,49,50].

In examined nut samples (except walnuts and Brazil nuts), the presence of CAPE was detected in concentration from 2.959 µg/g (pecan nuts) to trace amounts in pistachio nuts (0.012 µg/g), as showed in Table 2. CAPE is an ester of caffeic acid with multiple pharmacological activities, including great therapeutic effect in various types of cancer and protective effects on nervous system [51,52].

Literature data report large diversity in concentration of phenolic compounds, including flavonoids and aromatic acids among various types of nuts [3,29]. The most frequently identified flavonoids in nuts are catechin and epicatechin, which have been identified in hazelnuts, almonds, peanuts, pecans, walnuts and pine nuts [27,28,53,54,55,56]. In tested nuts, catechin was not identified in pecans, walnuts, hazelnuts, Brazil and macadamia nuts, while epicatechin was not detected only in almonds.

Pecans were characterized by the greatest variety of determined flavonoids among all tested nuts. Literature data indicate that pecans from Mexico contained ellagic acid, gallic acid, protocatechuic acid, p-hydroxybenzoic acid and catechin, and nuts form USA included gallic acid, catechin and ellagic acid; however, content of these phenols varied depending on cultivars [9,28,57]. The presence of caffeic and coumaric acids, which are also found in the tested nuts, was confirmed in three varieties of Texas pecans [28]. Additionally, in pecans from Turkey, the presence of syringic acid, quercetin, naringenin and kaempferol was confirmed, similar to their presence in the tested nuts [58]. In various cultivars of hazelnuts from different countries (including France, Hungary or Spain) catechin (in concentration range 1.9–26.3 µg/g) was detected in contrast to the tested nuts and the presence of epicatechin was confirmed (in concentration range 0.2–7.9 µg/g), similar to analyzed hazelnuts [27]. Moreover, according to the literature data, naringenin, quercetin, kaempferol and syringic acid were identified in hazelnuts, as well as, contrary to the analyzed nuts, caffeic, coumaric and vanillic acids [10,59,60]. The examined walnuts were characterized by a high concentration of epicatechin (114.296 µg/g) and content of catechin under detection limit. In turn, in walnuts from USA catechin was determined only in three from twelve tested cultivars in a concentration range 0.59–47.91 µg/g, while epicatechin was identified in ten from twelve cultivars in a concentration range 1.95–13.22 µg/g [55]. According to literature reports, ferulic, caffeic and cinnamic acids were detected in walnuts, which were also determined in the examined nuts, as well as sinapic, vanillic and syringic acids, rutin and naringenin, which were not detected in the nuts from this research [61]. Moreover, literature data indicate that, among flavonoids, naringenin was determined in almonds and pistachios, quercetin in Brazil nuts, pistachios, cashews, pine nuts and peanuts, rutin in almonds, taxifolin in pine nuts, kaempferol in cashews and peanuts, genistein in pistachios and apigenin in almonds. In turn, vanillic acid was identified in almonds and Brazil nuts; cinnamic acid in Brazil nuts and cashews; coumaric acid in Brazil nuts, cashews and peanuts; syringic acid in Brazil nuts, cashews and pine nuts; and gallic and ellagic acids were determined in Brazil nuts, peanuts, cashews and pine nuts [14,29,53,54,56,62,63,64,65,66]. Moreover, in most of the tested nuts, the presence of CAPE was found, a significant concentration of which was determined for pecans and peanuts. To the best of our knowledge, the presence of CAPE in edible nuts has not been previously reported.

### 2.2. Concentration of Mineral Elements

In tested nuts, the content of macro-elements and trace minerals was analyzed, and the results are presented in Table 3 and Table 4, respectively. The concentration of molybdenum and lead in all tested nut samples was under detection limit of atomic absorption spectrometry.

The content of macro-elements varied considerably among the type of tested nuts. Generally, the highest content of macro-elements was found in almonds (13,111.60 µg/g). The highest concentration of Ca was determined in almonds (1650.14 µg/g), Mg in Brazil nuts (5157.08 µg/g) and K in pistachios (6512.42 µg/g), while the highest amount of Na was found in hazelnuts (2861.86 µg/g) and pine nuts (2876.45 µg/g). The lowest micro-elements content was found in pecans and macadamia nuts. Macadamia nuts were characterized by the lowest concentration of potassium, while pecans contained the lowest content of calcium and magnesium among all tested nuts.

The concentration of Ca in tested nuts, except almonds, was lower than in nut samples from various countries, including Serbia, RPA, Chile, USA or Brazil [1,18,21,67,68,69]. The calcium content in tested almonds was higher in nuts from Serbian market (1491–1506 µg/g) and lower than in samples from RPA (5392.4 µg/g) and USA (3550 µg/g) [1,18,67]. The magnesium content in examined cashews (3449.93 µg/g), peanuts (2307.71 µg/g), hazelnuts (3168.56 µg/g) and pine nuts (4372.49 µg/g) was higher than in nuts from Serbian market (cashews, 2297–2444 µg/g; hazelnuts, 1497–1524 µg/g; peanuts, 2036–2079 µg/g) and from Brazilian pine nuts with Mg content equal 513 µg/g [18,68]. In contrast, the Mg content in macadamia nuts, pecans and pistachios from Polish market was lower than it concentration in nuts described in literature data [1,18]. The examined almonds, Brazil nuts and walnuts were characterized by higher Mg content compared to nuts from Serbian market and lower than examined in nuts from RPA [1,18]. The concentration of sodium and potassium in examined nuts varied depending on nut type, which confirmed statistical analysis. Literature data also confirms the large variation in the content of these macronutrients in the samples of nuts from different region of the world. The Na content was in a range from 50.8 µg/g for Brazilian pine nuts to 6848 µg/g for Serbian peanuts, while K concentration was determined in a range from 37.52 µg/g (peanuts from Serbia) to 8704.6 µg/g (Brazilian pine nuts) [18,68,70].

The concentration of trace elements in tested nuts varied depending on their type. Generally, the highest content of trace elements was found in pine nuts (192.79 µg/g) while the lowest concentration was determined for pistachios (93.24 mg/g). The Zn content in examined nuts was in a range from 22.87 µg/g (macadamia nuts) to 73.24 µg/g (pine nuts). The high amount of zinc was previously found in pecans (137.86 µg/g) and Brazil nuts (110.31 µg/g), while the low it content was confirmed in almonds (33.9 µg/g), hazelnuts (32.2 µg/g), peanuts (26.5 µg/g) and walnuts (22.6 µg/g) [1,12,71]. Copper was characterized by wide concentration variation among the type of nut samples analyzed, with concentration ranging from 7.41 µg/g (macadamia nuts) to 23.08 µg/g (cashews). According to literature data, the content of Cu in macadamia nuts ranged 3.41–18.96 µg/g, while Cu amount in cashews was in a range 23.08–28.57 µg/g [1,18,67]. Moreover, the high Cu content was found in walnuts (59.14 µg/g), hazelnuts (59.40 µg/g) and Brazil nuts (59.44 µg/g) [1,71]. The Mn content in examined nuts was determined in range from 10.89 µg/g for pine nuts to 54.93 µg/g for hazelnuts. In general, manganese was determined in different samples of nuts in various concentrations, including the content in macadamia nuts (88.64 µg/g), pecans (192.60 µg/g), almonds (3.9 µg/g) and Brazil nuts (only 3.40 µg/g) [1,12,18]. Large diversity in the Mn content in nut samples was observed by Wuillound et al., where content of this micro-elements ranged from 9 µg/g (cashews) to 4780 µg/g (almonds) [72]. Cashews were characterized by the highest Ni content (17.98 µg/g), while peanuts by the lowest Ni amount (8.80 µg/g) among all examined nuts. The nickel concentration determined in tested nuts was higher than in nut samples described in literature data, which was in a content range from 0.10 µg/g (pine nuts) to 7.33 µg/g (cashews) [18,73,74]. In turn, Wuillound et al. analyzed nine different type of nuts and stated high content of trace elements, such as Cu ranging from 186 µg/g (cashews) to 309 µg/g (pine nuts), Ni in concentration range from 44 µg/g (pine nuts) to 141 µg/g (cashews) and Zn content in a range from 189 µg/g (black walnuts) to 671 µg/g (pine nuts) [72]. The concentration of iron determined in tested nuts was in a range from 32.41 µg/g for macadamia nuts to 76.53 µg/g for pine nuts. According to literature reports, the Fe content in edible nuts was in a range from 19.60 µg/g (pine nuts) to 105.86 µg/g (pecans) [1,74]. However, the most frequently the Fe concentration in various nut samples was in the range 40–80 µg/g, and at this content level was determined in almonds, Brazil nuts, cashews, pistachios, walnuts and macadamia nuts [1,12,18,68]. In tested nuts, selenium was determined in all samples in trace amounts (0.88–1.14 µg/g), except for Brazil nuts, which had a higher Se content (11.27 µg/g). Trace content of selenium was also reported in almonds, walnuts, pistachios, hazelnuts and cashews [1,18,67,71]. In turn, the Se content in Brazil nuts was in a range from 0.7635 µg/g (nuts from Serbian market) to 38.7 µg/g (nuts from Northeast Brazil) [18,73]. Moreover, the presence of Cr was confirmed in trace amounts in Brazil nuts and pistachios, while Co in a concentration range 0.05–1.80 µg/g was determined in macadamia nuts, hazelnuts, almonds, pistachios, pine and Brazil nuts. Trace amounts of Co were also reported in Brazil nuts, cashews, peanuts, almonds, hazelnuts and pistachios, while trace content of Cr was determined in almonds, Brazil nuts, peanuts, macadamia nuts and pecans [1,12,18,74].

## 3. Materials and Methods

### 3.1. Nut Samples

The material used in the research was nine different types of edible nuts and peanuts available on the Polish market. Samples included raw pecans (*Carya illinoinensis*), toasted peanuts (*Arachis hypogaea*), raw walnuts (*Juglans regia*), raw cashews (*Anacardium occidentale*), raw macadamia nuts (*Macadamia tetraphylla*), raw hazelnuts (*Castanea sativa*), raw almonds (*Prunus amygdalus*), raw pine nuts (*Genus pine*), raw Brazil nuts (*Bertholletia excels*) and toasted pistachios (*Pistacia vera*). All nut samples were purchased from a local store with healthy food. After shell removal, the nut samples were ground in a Fritsch type 15 laboratory mill (IKA Werke, Staufen im Breisgau, Germany).

### 3.2. Ultra-High Pressure Liquid Chromatography (UPLC)

The ground nut samples (5 g) were added to 15 mL of methanol (Sigma-Aldrich, Steinheim, Germany) and sonicated for 1 h in temperature 25 °C. The nut extracts were centrifuged (Universal 320, Andreas Hettich GmbH & Co. KG, Tuttlingen, Germany) at 6000 rpm for 10 min at room temperature, and the supernatants were collected. The supernatants were mixed with 10 mL of n-hexane (Sigma-Aldrich, Steinheim, Germany) for 3 min in a vortex apparatus. Next, the methanol and hexane layers were separated, and the procedure was repeated three times with fresh portion of n-hexane. The methanol extracts were concentrated in a rotary evaporator (Buchi Labortechnik AG, Flawil, Switzerland) at 50 °C. The dry residue was then dissolved in methanol and analyzed on UPLC based on method described in literature [75].

The concentration of phenolic compounds (apigenin, catechin, epicatechin, rutin, myricetin, pinocembrin, pinobanksin, pinostrobin, naringenin, quercetin, galangin, kaempferol, CAPE, caffeic acid, ferulic acid, vanillic acid, syringic acid, sinapic acid, coumaric acid, hydroxycinnamic acid, cinnamic acid) was determined using the Aquity UPLC chromatograph (Waters, Manchester, MA, USA) equipped with a photodiode detector (PDA eλ Detector) (Waters, Manchester, MA, USA) and coupled to an electrospray ionization triple-quadrupole mass spectrometer (TQD) (Waters, Manchester, MA, USA). The nut extracts were filtered through a 0.20 µm syringe filter (Chromafil, Macherey-Nagel, Duren, Germany) before analyses. The compounds were separated at 25 °C on a Waters ACQUITY UPLC HSS T3 column (150 mm × 2.1 mm/ID, with 1.8 µm particle size) (Waters, Manchester, MA, USA). Gradient elution was applied using water containing 0.1% HCOOH (A) and acetonitrile containing 0.1% HCOOH (B) with the flow rate of 300 µL/min. The solvent gradient was modified as follows: 0–5 min 25% B, 5–20 min 40% B, 20–30 min 60% B, 30–35 min 90% B, and 35–40 min 100% B, followed by a return to the initial conditions. All samples were injected in triplicate. The phenolic compounds present in the samples were characterized according to their UV–Vis spectra and identified by their mass spectra and retention times in comparison with those of commercial standards. Limit of quantitation (LOQ) of the analytes was assessed by comparing the peak height to the baseline noise. The ratio of the peak height to baseline noise (signal-to-noise ratio) used for the estimation of the quantitation and detection limit were 10:1 and 3:1, respectively. The limit of detection (LOD) (in ng/g) for individual compounds was 1.5 (caffeic acid, sinapic acid, ferulic acid, quercetin, galangin), 3.0 (coumaric acid, hydroxycinnamic acid, cinnamic acid), 4.5 (CAPE, pinocembrin, apigenin, kaempferol, naringenin, vanillic acid, syringic acid), 6.0 (rutin, pinostrobin), 8.0 (catechin, pinobanksin) and 12.0 (epicatechin, myricetin). The percent relative standard deviation (%RSD) of the repeatability of the method, determined according to the peak area, did not exceed 3.97%. The obtained results confirm that this method can be used for the quantitative analysis of phenolic compounds.

### 3.3. Atomic Absorption Spectrometry (AAS)

The ground nut samples (0.5 g) were mineralized in nitric acid (8 mL) (Sigma-Aldrich, Steinheim, Germany) using the Mars Xpress mineralization system (CEM International Corporations, Matthews, NC, USA), following the three-step program (400 W, 100 °C for 2 min, 600 W, 160 °C for 5 min and 1600 W, 200 °C for 10 min). The digested solutions were filtered using filter paper and diluted to 50 mL with deionized water. This procedure was repeated three times for each sample.

The concentration of 14 elements, namely zinc (Zn), copper (Cu), manganese (Mn), magnesium (Mg), cobalt (Co), potassium (K), sodium (Na), iron (Fe), selenium (Se), calcium (Ca), lead (Pb), nickel (Ni), chrome (Cr) and molybdenum (Mo) in nut samples was analyzed according to the method described in the literature [76]. The determination was performed using an AA280 FS/Z AA spectrometer (Agilent Technologies, Santa Clara, CA, USA) equipped with a single-element hollow cathode lamp. The calibration curve for each element was prepared with five replicates based on a series of freshly prepared standard solutions obtained from stock standards (Sigma-Aldrich, Steinheim, Germany). The results were expressed as the average values from three simultaneous measurements.

### 3.4. Statistical Analysis

Statistical analyses included factorial one-way ANOVA, followed by Tukey’s honest significant difference (HSD) test at α = 0.05. All the statistical analyses were performed using the TIBCO Software Inc. Statistica version 13.1 (Palo Alto, CA, USA).

## 4. Conclusions

The study presents the phenolic profile and mineral content in nine commonly consumed tree nuts and peanuts available on the Polish market. The phenolic profile of examined nuts was significantly varied depending on the type of nut. Pecans were characterized by the greatest diversity of individual flavonoids, containing nine out twelve analyzed compounds. In contrast, only three flavonoids were identified in Brazil nuts—epicatechin, pinostrobin and galangin. The highest total content of all analyzed flavonoids was determined in walnuts (114.861 µg/g), while the lowest in almonds (1.717 µg/g). Epicatechin was the most abundant flavonoids in nuts, except almonds, which did not it contain. Galangin was the only flavonoid identified in all examined nuts, with concentration range from 0.007 µg/g (pistachios) to 2.276 µg/g (macadamia nuts). In turn, sinapic acid was the most frequently identified aromatic acid in tested nuts. It was detected in all nut samples (except walnuts) in a concentration range from 0.050 µg/g (macadamia nuts) to 10.373 µg/g (pecans). The highest total content of all tested aromatic acid was determined in pecans (33.743 µg/g), while the lowest in almonds (0.096 µg/g). In addition, in almonds, only three aromatic acids were identified: caffeic, sinapic and ferulic. The cinnamic acid was detected in the highest concentration among all analyzed aromatic acids, and in pecans its concentration was 12.774 µg/g. Moreover, in examined nuts (except walnuts and Brazil nuts), the presence of CAPE, which exhibits multiple biological properties, was confirmed. The content of mineral elements in nuts varied considerably among the type of tested nuts. The highest content of macro-elements was found in almonds (13,111.60 µg/g), while the lowest in pecans (5425.39 µg/g) and macadamia nuts (6446.19 µg/g). In turn, the highest content of trace elements was determined in pine nuts (192.79 µg/g), while the lowest were in pistachios (93.24 µg/g). Moreover, the content of lead and molybdenum in all tested nuts was below the detection limit of AAS.

The obtained results indicate that the tested nuts are characterized by a significant diversity in the content of both phenolic compounds and minerals. However, all types of nuts, apart from the well-known source of fatty acids, are a rich source of various components with beneficial effect on human health. The results presented in this study could expand the existing knowledge of the chemical composition and presence of phytochemicals in nuts. The results may also be of interest to various groups of scientists, including biochemists, food scientists and nutritionists. In addition, the article can provide consumers with valuable information that can help them choose a specific type of nut, not only taking into account the taste but also the properties of their ingredients.

## Figures and Tables

**Table 1:** The concentration of flavonoids in nuts.

Concentration (µg/g Fresh Weight)	Pecan	Peanut	Walnut	Cashew	Macadamia	Hazelnut	Almond	Pine	Brazil	Pistachio
Catechin	nd	8.072 ^b^ ± 0.131	nd	22.263 ^a^ ± 0.249	1.063 ^c^ ± 0.057	nd	0.722 ^c^ ± 0.056	0.947 ^c^ ± 0.061	nd	2.216 ^c^ ± 0.229
Epicatechin	81.582 ^b^ ± 5.954	4.843 ^b^ ± 0.164	114.296 ^a^ ± 20.867	24.173 ^b^ ± 3.044	4.885 ^b^ ± 0.111	5.625 ^b^ ± 0.369	nd	1.162 ^b^ ± 0.085	7.280 ^b^ ± 0.215	2.405 ^b^ ± 0.138
Rutin	nd	nd	nd	0.130 ± 0.007	nd	nd	0.110 ± 0.009	nd	nd	nd
Myricetin	1.230 ± 0.081	nd	nd	nd	nd	nd	nd	nd	nd	1.196 ± 0.105
Pinobanksin	nd	0.446 ^b^ ± 0.042	nd	nd	0.031 ^d^ ± 0.007	0.188 ^c^ ± 0.017	nd	nd	nd	0.637 ^a^ ± 0.037
Naringenin	0.116 ^c^ ± 0.011	nd	nd	0.021 ^d^ ± 0.005	nd	0.370 ^b^ ± 0.027	0.496 ^a^ ± 0.021	nd	nd	nd
Quercetin	0.065 ^b^ ± 0.003	nd	nd	nd	nd	0.027 ^b,c^ ± 0.004	0.010 ^c^ ± 0.002	0.065 ^b^ ± 0.005	nd	0.641 ^a^ ± 0.037
Pinocembrin	0.055 ± 0.005	nd	nd	nd	nd	nd	nd	0.069 ± 0.006	nd	nd
Apigenin	0.074 ^a^ ± 0.005	nd	0.027 ^c^ ± 0.004	0.046 ^b^ ± 0.005	0.019 ^c^ ± 0.003	0.020 ^c^ ± 0.005	nd	0.031 ^c^ ± 0.004	nd	nd
Kaempferol	0.282 ^a^ ± 0.018	0.115 ^b^ ± 0.008	nd	0.010 ^e^ ± 0.003	0.017 ^e^ ± 0.002	0.042 ^d^ ± 0.003	0.007 ^e^ ± 0.001	0.071 ^c^ ± 0.002	nd	0.006 ^e^ ± 0.001
Pinostrobin	6.335 ^a^ ± 0.316	nd	0.484 ^b^ ± 0.021	0.352 ^b,c^ ± 0.016	0.388 ^b^ ± 0.022	0.206 ^b,c^ ± 0.016	0.361 ^b^ ± 0.019	0.257 ^b,c^ ± 0.012	0.312 ^b,c^ ± 0.009	nd
Galangin	1.169 ^b^ ± 0.063	0.227 ^c^ ± 0.021	0.054 ^c^ ± 0.005	0.010 ^c^ ± 0.002	2.276 ^a^ ± 0.247	0.018 ^c^ ± 0.001	0.011 ^c^ ± 0.001	1.215 ^b^ ± 0.108	0.035 ^c^ ± 0.004	0.007 ^c^ ± 0.001
*Sum of flavonoids*	*90.908*	*13.703*	*114.861*	*47.005*	*8.679*	*6.496*	*1.717*	*77.057*	*58.307*	*31.738*

Expressed as average ± standard deviations. Values denoted with identical letters do not differ significantly; nd—the content under detection limit of UPLC.

**Table 2:** The concentration of phenolic acids and CAPE in nuts.

Concentration (µg/g Fresh Weight)	Pecan	Peanut	Walnut	Cashew	Macadamia	Hazelnut	Almond	Pine	Brazil	Pistachio
Vanillic acid	1.040 ^c^ ± 0.055	0.951 ^c,d^ ± 0.080	nd	nd	3.164 ^a^ ± 0.144	nd	nd	2.154 ^b^ ± 0.179	0.684^d^ ± 0.023	0.158 ^e^ ± 0.008
Syringic acid	6.581 ^a,b^ ± 0.176	7.404 ^a^ ± 0.336	nd	4.285 ^d^ ± 0.367	3.249 ^e^ ± 0.688	0.766 ^f^ ± 0.041	nd	5.389 ^c^ ± 0.168	nd	5.768 ^b,c^ ± 0.219
Caffeic acid	2.950 ^b^ ± 0.153	nd	5.280 ^a^ ± 0.227	0.012 ^d^ ± 0.002	0.240 ^d^ ± 0.015	nd	0.050 ^d^ ± 0.003	0.166 ^d^ ± 0.027	1.124 ^c^ ± 0.117	nd
Sinapic acid	10.373 ^a^ ± 0.817	0.648 ^c^ ± 0.040	nd	0.125 ^c^ ± 0.035	0.050 ^c^ ± 0.004	3.431 ^b^ ± 0.373	0.016 ^c^ ± 0.004	0.096 ^c^ ± 0.012	0.124 ^c^ ± 0.010	0.282 ^c^ ± 0.020
Coumaric acid	0.055 ^d^ ± 0.004	0.965 ^b^ ± 0.043	1.265 ^a^ ± 0.064	0.018 ^d^ ± 0.002	nd	nd	nd	nd	nd	0.499 ^c^ ± 0.020
Hydroxycinnamic acid	nd	nd	nd	nd	nd	0.318 ± 0.014	nd	0.295 ± 0.016	nd	nd
Ferulic acid	nd	0.141 ^b^ ± 0.010	1.648 ^a^ ± 0.057	0.002 ^c^ ± 0.000	nd	nd	0.030 ^c^ ± 0.002	nd	nd	0.112 ^b^ ± 0.010
Cinnamic acid	12.774 ^a^ ± 2.240	0.493 ^b^ ± 0.012	0.716 ^b^ ± 0.024	0.126 ^b^ ± 0.007	0.055 ^b^ ± 0.006	0.055 ^b^ ± 0.005	nd	nd	nd	0.114 ^b^ ± 0.012
*Sum of phenolic acids*	*33.743*	*10.602*	*8.909*	*4.568*	*6.758*	*4.570*	*0.096*	*8.100*	*1.932*	*6.933*
CAPE	2.959 ^a^ ± 0.129	2.135 ^b^ ± 0.144	nd	0.108 ^c^ ± 0.007	0.153 ^c^ ± 0.015	0.056 ^c^ ± 0.005	0.090 ^c^ ± 0.013	0.114 ^c^ ± 0.013	nd	0.012 ^c^ ± 0.002

Expressed as average ± standard deviations. Values denoted with identical letters do not differ significantly; nd—the content under detection limit of UPLC.

**Table 3:** The content of macro-elements in nuts.

Concentration (µg/g Fresh Weight)	Pecan	Peanut	Walnut	Cashew	Macadamia	Hazelnut	Almond	Pine	Brazil	Pistachio
Ca	388.84 ^g^ ± 0.46	412.32 ^g^ ± 2.55	728.69 ^e^ ± 18.63	289.99 ^h^ ± 1.52	464.99 ^f^ ± 2.23	1218.96 ^b^ ± 12.37	1650.14 ^a^ ± 19.86	216.40 ^i^ ± 5.03	1029.52 ^c^ ± 12.50	873.01 ^d^ ± 7.84
Mg	1568.10 ^g^ ± 7.38	2309.71 ^e^ ± 2.64	2346.47 ^e^ ± 67.65	3449.93 ^c^ ± 24.40	1881.94 ^f^ ± 37.74	3168.56 ^d^ ± 21.72	4206.70 ^b^ ± 40.79	4372.49 ^b^ ± 126.19	5157.08 ^a^ ± 77.94	1427.22 ^g^ ± 50.74
K	2974.32 ^h^ ± 28.86	5405.35 ^b,c^ ± 37.53	3664.43 ^g^ ± 50.12	4996.21 ^e^ ± 38.18	2473.88 ^i^ ± 26.15	5533.51 ^b^ ± 33.38	5157.81 ^d^ ± 15.20	5349.90 ^c^ ± 33.07	4302.67 ^f^ ± 20.44	6512.42 ^a^ ± 71.48
Na	494.13 ^g^ ± 7.71	1110.56 ^f^ ± 16.12	285.50 ^h^ ± 16.19	1160.56 ^f^ ± 7.92	1625.38 ^d^ ± 15.72	2861.86 ^a^ ± 14.69	2096.95 ^c^ ± 20.24	2876.45 ^a^ ± 26.19	1429.01 ^e^ ± 12.49	2446.28 ^b^ ± 10.06
*Sum of macro-elements*	*5425.39*	*9237.94*	*7025.09*	*9896.69*	*6446.19*	*12,782.89*	*13,111.60*	*12,815.24*	*11,918.28*	*11,258.93*

Expressed as average ± standard deviations. Values denoted with identical letters do not differ significantly.

**Table 4:** The content of trace elements in nuts.

Concentration (µg/g Fresh Weight)	Pecan	Peanut	Walnut	Cashew	Macadamia	Hazelnut	Almond	Pine	Brazil	Pistachio
Zn	59.90 ^b^ ± 0.78	39.02 ^e^ ± 0.61	39.47 ^e^ ± 0.49	56.42 ^c^ ± 0.49	22.87 ^h^ ± 0.18	30.69 ^f^ ± 0.15	37.82 ^e^ ± 0.11	73.24 ^a^ ± 0.79	50.68 ^d^ ± 0.15	24.63 ^g^ ± 0.12
Cu	13.56 ^d^ ± 0.36	9.52 ^e,f^ ± 0.18	10.16 ^e^ ± 0.15	23.08 ^a^ ± 0.54	7.41 ^g^ ± 0.21	16.80 ^c^ ± 0.15	9.89 ^e^ ± 0.19	16.40 ^c^ ± 0.21	19.88 ^b^ ± 0.17	8.98 ^f^ ± 0.08
Mn	34.44 ^c^ ± 0.19	17.80 ^f^ ± 0.34	45.08 ^b^ ± 0.20	21.55 ^e^ ± 0.13	44.40 ^b^ ± 0.43	54.93 ^a^ ± 0.53	25.79 ^d^ ± 0.33	10.89 ^h^ ± 0.38	13.25 ^g^ ± 0.09	12.81 ^g^ ± 0.15
Ni	13.34 ^b,c^ ± 0.39	8.80 ^f^ ± 0.18	11.76 ^d,e^ ± 0.50	17.98 ^a^ ± 0.23	10.65 ^e^ ± 0.39	14.40 ^b^ ± 0.80	12.57 ^c,d^ ± 0.30	14.22 ^b^ ± 0.49	16.98 ^a^ ± 0.20	13.26 ^b,c^ ± 0.24
Se	1.14 ^b^ ± 0.01	0.98 ^b^ ± 0.01	1.08 ^b^ ± 0.03	0.87 ^b^ ± 0.01	0.97 ^b^ ± 0.04	0.88 ^b^ ± 0.16	0.98 ^b^ ± 0.03	1.04 ^b^ ± 0.01	11.27 ^a^ ± 0.52	1.10 ^b^ ± 0.02
Fe	39.39 ^d^ ± 2.18	31.92 ^f^ ± 3.64	37.80 ^d,e^ ± 1.14	68.78 ^b^ ± 0.52	32.41 ^f^ ± 0.45	41.12 ^d^ ± 0.03	50.78 ^c^ ± 0.30	76.53 ^a^ ± 0.79	33.53 ^e,f^ ± 0.14	30.27 ^f^ ± 0.55
Cr	nd	nd	nd	nd	nd	nd	nd	nd	0.55 ± 0.42	1.32 ± 0.37
Co	nd	nd	nd	nd	0.27 ^d^ ± 0.03	0.05 ± 0.01	0.25 ^c.d^ ± 0.10	0.47 ^c^ ± 0.14	1.80 ^a^ ± 0.18	0.87 ^b^ ± 0.09
*Sum of trace elements*	*161.77*	*108.04*	*145.35*	*188.68*	*118.98*	*158.87*	*138.08*	*192.79*	*147.94*	*93.24*

Expressed as average ± standard deviations. Values denoted with identical letters do not differ significantly; nd—the content under detection limit of AAS.

## Data Availability

The data reported in this study are available on request from the authors.

## Sample Availability

Samples of the compounds are available from the authors.

## References

[1] Moodley R., Kindness A., Jonnalagadda S.B. (2007). Elemental Composition and Chemical Characteristics of Five Edible Nuts (Almond, Brazil, Pecan, Macadamia and Walnut) Consumed in Southern Africa. J. Environ. Sci. Health Part B Pestic. Food Contam. Agric. Wastes.

[2] De Souza R.G.M., Schincaglia R.M., Pimente G.D., Mota J.F. (2017). Nuts and Human Health Outcomes: A Systematic Review. Nutrients.

[3] Taş N.G., Gökmen V. (2017). Phenolic Compounds in Natural and Roasted Nuts and Their Skins: A Brief Review. Curr. Opin. Food Sci..

[4] Alasalvar C., Salvadó J.S., Ros E. (2020). Bioactives and Health Benefits of Nuts and Dried Fruits. Food Chem..

[5] Coates A., Hill A., Tan S. (2018). Nuts and Cardiovascular Disease Prevention. Curr. Atheroscler. Rep..

[6] Stevens-Barrón J.C., Mizerska-Kowalska M., Płazí W., Sowa S., Paduch R., Grumezescu A.M., Stevens-Barrón J.C., Wall-Medrano A., Álvarez-Parrilla E., Olivas-Armendáriz I. (2022). Synergistic Interactions between Tocol and Phenolic Extracts from Different Tree Nut Species against Human Cancer Cell Lines. Molecules.

[7] Yang J., Liu R.H., Halim L. (2009). Antioxidant and Antiproliferative Activities of Common Edible Nut Seeds. LWT—Food Sci. Technol..

[8] Gorji N., Moeini R., Memariani Z. (2018). Almond, Hazelnut and Walnut, Three Nuts for Neuroprotection in Alzheimer’s Disease: A Neuropharmacological Review of Their Bioactive Constituents. Pharmacol. Res..

[9] Flores-Córdova M.A., Sánchez E., Muñoz-Márquez E., Ojeda-Barrios D.L., Soto-Parra J.M., Preciado-Rangel P. (2017). Phytochemical Composition and Antioxidant Capacity in Mexican Pecan Nut. Emir. J. Food Agric..

[10] Kit W.S., Priya M., Chin J.H., Mariam A., Akowuah G.A. (2015). Antimicrobial and Antiradical Activities of *Corylus cornuta* (Marsh. Betulacea) Kernel Extracts. Orient. Pharm. Exp. Med..

[11] de Camargo A.C., Regitano-d’Arce M.A.B., Rasera G.B., Canniatti-Brazaca S.G., do Prado-Silva L., Alvarenga V.O., Sant’Ana A.S., Shahidi F. (2017). Phenolic Acids and Flavonoids of Peanut By-Products: Antioxidant Capacity and Antimicrobial Effects. Food Chem..

[12] Momen A.A., Zachariadis G.A., Anthemidis A.N., Stratis J.A. (2007). Use of Fractional Factorial Design for Optimization of Digestion Procedures Followed by Multi-Element Determination of Essential and Non-Essential Elements in Nuts Using ICP-OES Technique. Talanta.

[13] Lin J.T., Liu S.C., Hu C.C., Shyu Y.S., Hsu C.Y., Yang D.J. (2016). Effects of Roasting Temperature and Duration on Fatty Acid Composition, Phenolic Composition, Maillard Reaction Degree and Antioxidant Attribute of Almond (*Prunus dulcis)* Kernel. Food Chem..

[14] Uslu N., Özcan M.M. (2019). Effect of Microwave Heating on Phenolic Compounds and Fatty Acid Composition of Cashew (*Anacardium occidentale*) Nut and Oil. J. Saudi Soc. Agric. Sci..

[15] Kornsteiner-Krenn M., Wagner K.H., Elmadfa I. (2014). Phytosterol Content and Fatty Acid Pattern of Ten Different Nut Types. Int. J. Vitam. Nutr. Res..

[16] Ros E. (2010). Health Benefits of Nut Consumption. Nutrients.

[17] Venkatachalan M., Sathe S.K. (2006). Chemical Composition of Selected Edible Nut Seeds. J. Agric. Food Chem..

[18] Tošić S.B., Mitić S.S., Velimirović D.S., Stojanović G.S., Pavlović A.N., Pecev-Marinković E.T. (2015). Elemental Composition of Edible Nuts: Fast Optimization and Validation Procedure of an ICP-OES Method. J. Sci. Food Agric..

[19] Siddiqui K., Bawazeer N., Scaria Joy S. (2014). Variation in Macro and Trace Elements in Progression of Type 2 Diabetes. Sci. World J..

[20] Fraga C.G. (2005). Relevance, Essentiality and Toxicity of Trace Elements in Human Health. Mol. Asp. Med..

[21] Cardoso B.R., Duarte G.B.S., Reis B.Z., Cozzolino S.M.F. (2017). Brazil Nuts: Nutritional Composition, Health Benefits and Safety Aspects. Food Res. Int..

[22] Albuquerque B.R., Heleno S.A., Oliveira M.B.P.P., Barros L., Ferreira I.C.F.R. (2021). Phenolic Compounds: Current Industrial Applications, Limitations and Future Challenges. Food Funct..

[23] Tuli H.S., Sak K., Adhikary S., Kaur G., Aggarwal D., Kaur J., Kumar M., Parashar N.C., Parashar G., Sharma U. (2022). Galangin: A Metabolite That Suppresses Anti-Neoplastic Activities through Modulation of Oncogenic Targets. Exp. Biol. Med..

[24] Caleja C., Ribeiro A., Barreiro M.F., Ferreira I.C.F.R. (2017). Phenolic Compounds as Nutraceuticals or Functional Food Ingredients. Curr. Pharm. Des..

[25] Hama J.R., Omer R.A., Rashid R.S.M., Mohammad N.-E.-A., Thoss V. (2016). The Diversity of Phenolic Compounds along Defatted Kernel, Green Husk and Leaves of Walnut (*Juglans regia* L.). Let. Org. Chem..

[26] Durazzo A., Lucarini M., Souto E.B., Cicala C., Caiazzo E., Izzo A.A., Novellino E., Santini A. (2019). Polyphenols: A Concise Overview on the Chemistry, Occurrence, and Human Health. Phyther. Res..

[27] Jakopic J., Petkovsek M.M., Likozar A., Solar A., Stampar F., Veberic R. (2011). HPLC–MS Identification of Phenols in Hazelnut (*Corylus avellana* L.) Kernels. Food Chem..

[28] Malik N.S.A., Perez J.L., Lombardini L., Cornacchia R., Cisneros-Zevallosb L., Braforda J. (2009). Phenolic Compounds and Fatty Acid Composition of Organic and Conventional Grown Pecan Kernels. J. Sci. Food Agric..

[29] Bodoira R., Maestri D. (2020). Phenolic Compounds from Nuts: Extraction, Chemical Profiles, and Bioactivity. J. Agric. Food Chem..

[30] Fraga C.G., Oteiza P.I. (2011). Dietary Flavonoids: Role of (−)-Epicatechin and Related Procyanidins in Cell Signaling. Free Radic. Biol. Med..

[31] Qu Z., Liu A., Li P., Liu C., Xiao W., Huang J., Liu Z., Zhang S. (2020). Advances in Physiological Functions and Mechanisms of (−)-Epicatechin. Crit. Rev. Food Sci. Nutr..

[32] Prakash M., Basavaraj B.V., Chidambara Murthy K.N. (2019). Biological Functions of Epicatechin: Plant Cell to Human Cell Health. J. Funct. Foods.

[33] Chun J.H., Henckel M.M., Knaub L.A., Hull S.E., Pott G.B., Walker L.A., Reusch J.E.B., Keller A.C. (2022). (−)-Epicatechin Improves Vasoreactivity and Mitochondrial Respiration in Thermoneutral-Housed Wistar Rat Vasculature. Nutrients.

[34] Baranwal A., Aggarwal P., Rai A., Kumar N. (2021). Pharmacological Actions and Underlying Mechanisms of Catechin: A Review. Mini-Rev. Med. Chem..

[35] Ganeshpurkar A., Saluja A. (2020). The Pharmacological Potential of Catechin. Indian J. Biochem. Biophys..

[36] Sinsinwar S., Vadivel V. (2020). Catechin Isolated from Cashew Nut Shell Exhibits Antibacterial Activity against Clinical Isolates of MRSA through ROS-Mediated Oxidative Stress. Appl. Microbiol. Biotechnol..

[37] Fang D., Xiong Z., Xu J., Yin J., Luo R. (2019). Chemopreventive Mechanisms of Galangin against Hepatocellular Carcinoma: A Review. Biomed. Pharmacother..

[38] Singh D., Saini A., Singh R., Agrawal R. (2022). Galangin, as a Potential Anticancer Agent. Rev. Bras. Farmacogn..

[39] Biharee A., Sharma A., Kumar A., Jaitak V. (2020). Antimicrobial Flavonoids as a Potential Substitute for Overcoming Antimicrobial Resistance. Fitoterapia.

[40] Echeverría J., Opazo J., Mendoza L., Urzúa A., Wilkens M. (2017). Structure-Activity and Lipophilicity Relationships of Selected Antibacterial Natural Flavones and Flavanones of Chilean Flora. Molecules.

[41] Chudapongse N., Klahan K., Kamkhunthod M., Ratchawong C., Nantapong N. (2010). Antifungal Activity against *Candida albicans* and Effect on Mitochondrial NADH Oxidation of Galangin. Planta Med..

[42] Rafał I.G., Króliczewski B.J., Górniak I., Bartoszewski R., Króliczewski Á.J. (2018). Comprehensive Review of Antimicrobial Activities of Plant Flavonoids. Phytochem. Rev..

[43] Farhadi F., Khameneh B., Iranshahi M., Iranshahy M. (2019). Antibacterial Activity of Flavonoids and Their Structure–Activity Relationship: An Update Review. Phyther. Res..

[44] Patel N.K., Jaiswal G., Bhutani K.K. (2015). A Review on Biological Sources, Chemistry and Pharmacological Activities of Pinostrobin. Nat. Prod. Res..

[45] Nićiforović N., Abramovič H. (2014). Sinapic Acid and Its Derivatives: Natural Sources and Bioactivity. Compr. Rev. Food Sci. Food Saf..

[46] Nguyen V.P.T., Stewart J.D., Ioannou I., Allais F. (2021). Sinapic Acid and Sinapate Esters in Brassica: Innate Accumulation, Biosynthesis, Accessibility via Chemical Synthesis or Recovery from Biomass, and Biological Activities. Front. Chem..

[47] Szwajgier D., Borowiec K., Pustelniak K. (2017). The Neuroprotective Effects of Phenolic Acids: Molecular Mechanism of Action. Nutrients.

[48] Sevgi K., Tepe B., Sarikurkcu C. (2015). Antioxidant and DNA Damage Protection Potentials of Selected Phenolic Acids. Food Chem. Toxicol..

[49] Heleno S.A., Martins A., Queiroz M.J.R.P., Ferreira I.C.F.R. (2015). Bioactivity of Phenolic Acids: Metabolites versus Parent Compounds: A Review. Food Chem..

[50] Weng J.R., Lin C.S., Lai H.C., Lin Y.P., Wang C.Y., Tsai Y.C., Wu K.C., Huang S.H., Lin C.W. (2019). Antiviral Activity of Sambucus FormosanaNakai Ethanol Extract and Related Phenolic Acid Constituents against Human Coronavirus NL63. Virus Res..

[51] Tolba M.F., Azab S.S., Khalifa A.E., Abdel-Rahman S.Z., Abdel-Naim A.B. (2013). Caffeic Acid Phenethyl Ester, a Promising Component of Propolis with a Plethora of Biological Activities: A Review on Its Anti-Inflammatory, Neuroprotective, Hepatoprotective, and Cardioprotective Effects. IUBMB Life.

[52] Lv L., Cui H., Ma Z., Liu X., Yang L. (2021). Recent Progresses in the Pharmacological Activities of Caffeic Acid Phenethyl Ester. Naunyn-Schmiedeberg’s Arch. Pharmacol..

[53] Milbury P.E., Chen C.Y., Dolnikowski G.G., Blumberg J.B. (2006). Determination of Flavonoids and Phenolics and Their Distribution in Almonds. J. Agric. Food Chem..

[54] Abdul-Hamid A., Baharin B.S., Anwar F., Sabu M.C., Pak-Dek M.S. (2011). Phenolic Compounds and Antioxidant Activity of Peanut’s Skin, Hull, Raw Kernel and Roasted KErnel Flour. Pak. J. Bot.

[55] Vu D.C., Vo P.H., Coggeshall M.V., Lin C.H. (2018). Identification and Characterization of Phenolic Compounds in Black Walnut Kernels. J. Agric. Food Chem..

[56] Hoon L.Y., Choo C., Watawana M.I., Jayawardena N., Waisundara V.Y. (2015). Evaluation of the Total Antioxidant Capacity and Antioxidant Compounds of Different Solvent Extracts of Chilgoza Pine Nuts (*Pinus gerardiana*). J. Funct. Foods.

[57] De La Rosa L.A., Alvarez-Parrilla E., Shahidi F. (2010). Phenolic Compounds and Antioxidant Activity of Kernels and Shells of Mexican Pecan (*Carya illinoinensis*). J. Agric. Food Chem..

[58] Juhaimi F.A., Özcan M.M., Uslu N., Doğu S. (2017). Pecan Walnut (*Carya*
*illinoinensis* (Wangenh.) K. Koch) Oil Quality and Phenolic Compounds as Affected by Microwave and Conventional Roasting. J. Food Sci. Technol..

[59] Özcan M.M., Juhaimi F.A., Uslu N. (2017). The Effect of Heat Treatment on Phenolic Compounds and Fatty Acid Composition of Brazilian Nut and Hazelnut. J. Food Sci. Technol..

[60] Ciemniewska-Zytkiewicz H., Verardo V., Pasini F., Bryś J., Koczoń P., Caboni M.F. (2015). Determination of Lipid and Phenolic Fraction in Two Hazelnut (*Corylus avellana* L.) Cultivars Grown in Poland. Food Chem..

[61] Wu S., Shen D., Wang R., Li Q., Mo R., Zheng Y., Zhou Y., Liu Y. (2021). Phenolic Profiles and Antioxidant Activities of Free, Esterified and Bound Phenolic Compounds in Walnut Kernel. Food Chem..

[62] Tomaino A., Martorana M., Arcoraci T., Monteleone D., Giovinazzo C., Saija A. (2010). Antioxidant Activity and Phenolic Profile of Pistachio (*Pistacia vera* L., Variety Bronte) Seeds and Skins. Biochimie.

[63] Chandrasekara N., Shahidi F. (2011). Effect of Roasting on Phenolic Content and Antioxidant Activities of Whole Cashew Nuts, Kernels, and Testa. J. Agric. Food Chem..

[64] Zulfqar F., Akhtar M.F., Saleem A., Akhtar B., Sharif A., Saleem U. (2020). Chemical Characterization, Antioxidant Evaluation, and Antidiabetic Potential of *Pinus gerardiana* (Pine Nuts) Extracts. J. Food Biochem..

[65] John J.A., Shahidi F. (2010). Phenolic Compounds and Antioxidant Activity of Brazil Nut (*Bertholletia*
*excelsa*). J. Funct. Foods.

[66] Bolling B.W., Chen C.Y.O., McKay D.L., Blumberg J.B. (2011). Tree Nut Phytochemicals: Composition, Antioxidant Capacity, Bioactivity, Impact Factors. A Systematic Review of Almonds, Brazils, Cashews, Hazelnuts, Macadamias, Pecans, Pine Nuts, Pistachios and Walnuts. Nutr. Res. Rev..

[67] Schlörmann W., Birringer M., Böhm V., Löber K., Jahreis G., Lorkowski S., Müller A.K., Schöne F., Glei M. (2015). Influence of Roasting Conditions on Health-Related Compounds in Different Nuts. Food Chem..

[68] Silva E.F.R., da Silva Santos B.R., Minho L.A.C., Brandão G.C., de Jesus Silva M., Silva M.V.L., dos Santos W.N.L., dos Santos A.M.P. (2022). Characterization of the Chemical Composition (Mineral, Lead and Centesimal) in Pine Nut (*Araucaria*
*angustifolia* (Bertol.) Kuntze) Using Exploratory Data Analysis. Food Chem..

[69] Henríquez C., Loewe V., Saavedra J., Córdova A., Lutz M. (2018). Effect of the Type of Packaging on the Oxidative Stability of Pine Nuts (*Pinus pinea* L.) Grown in Chile. CYTA-J. Food.

[70] Lutz M., Álvarez K., Loewe V. (2016). Chemical Composition of Pine Nut (*Pinus*
*pinea* L.) Grown in Three Geographical Macrozones in Chile. CYTA-J. Food.

[71] Amini-Noori F., Ziarati P. (2015). Chemical Composition of Native Hazelnut (*Corylus*
*avellana* L.) Varieties in Iran, Association with Ecological Conditions. Biosci. Biotechnol. Res. Asia.

[72] Wuilloud R.G., Kannamkumarath S.S., Caruso J.A. (2004). Speciation of Nickel, Copper, Zinc, and Manganese in Different Edible Nuts: A Comparative Study of Molecular Size Distribution by SEC–UV–ICP–MS. Anal. Bioanal. Chem..

[73] Lopes G.S., Silva F.L.F., Grinberg A.P., Sturgeon R.E. (2016). An Evaluation of the Use of Formic Acid for Extraction of Trace Elements from Brazil Nut and Babassu Coconut and Its Suitability for Multi-Element Determination by ICP-MS. J. Braz. Chem. Soc..

[74] Cabrera C., Lloris F., Giménez R., Olalla M., López M.C. (2003). Mineral Content in Legumes and Nuts: Contribution to the Spanish Dietary Intake. Sci. Total Environ..

[75] Woźniak M., Mrówczyńska L., Kwaśniewska-Sip P., Waśkiewicz A., Nowak P., Ratajczak I. (2020). Effect of the Solvent on Propolis Phenolic Profile and Its Antifungal, Antioxidant, and In Vitro Cytoprotective Activity in Human Erythrocytes under Oxidative Stress. Molecules.

[76] Cvek J., Medić-Šarić M., Vitali D., Vedrina-Dragojević I., Šmit Z., Tomić S. (2008). The Content of Essential and Toxic Elements in Croatian Propolis Samples and Their Tinctures. J. Apic. Res..

